# Modeling the spatial and temporal dynamics of riparian vegetation induced by river flow fluctuation

**DOI:** 10.1002/ece3.3886

**Published:** 2018-03-05

**Authors:** Xiaoguang You, Jingling Liu

**Affiliations:** ^1^ State Key Joint Laboratory of Environmental Simulation and Pollution Control School of Environment Beijing Normal University Beijing China

**Keywords:** age structure, nonstationarity, population lifespan, riparian vegetation distribution, river flow fluctuation, same‐aged population, spatial temporal dynamics, species abundance, stochastic model

## Abstract

River flow fluctuation has an important influence on riparian vegetation dynamics. A temporally segmented stochastic model focusing on a same‐aged population is developed for the purpose of describing both spatial and temporal dynamics of riparian vegetation. In the model, the growth rate of population, rather than carrying capacity, is modeled as the random variable. This model has explicit physical meaning. The model deduces a process‐based solution. From the solution process, the probability density of spatial distribution can be derived; therefore, the spatial distribution of population abundance can be described. The lifespan of a same‐aged population and the age structure of the species‐specific population can also be studied with the aid of this temporally segmented model. The influence of correlation time of river flow fluctuation is also quantified according to the model. The calibration of model parameters and model application are discussed. The model provides a computer‐aided method to simulate and predict vegetation dynamics during river flow disturbances. Meanwhile, the model is open and allows for more accurate and concrete modeling of growth rate. Because of the Markov property involved in the process‐based solution, the model also has the ability to deal with cases of nonstationary disturbances.

## 1 INTRODUCTION

Riparian zones are transitional areas between terrestrial and aquatic ecosystems (Naiman, Décamps, & McClain, 2010; Roath & Krueger, 1982). These zones provide important ecosystem services, including nutrient cycling, flood attenuation, riverbank stabilization, water purification, groundwater recharge, and flow regulation (Kauffman, Beschta, Otting, & Lytjen, 1997; Meek, Richardson, & Mucina, 2010; Tickner, Angold, Gurnell, & Mountford, 2001), which are ultimately governed by the plant communities along the river. Riparian vegetation dynamics, including the recruitment, growth, reproduction, dispersal, and mortality of individual plants, are greatly influenced by flow regime alterations induced by natural or anthropogenic interferences (Auble, Friedman, & Scott, 1994; Bendix & Hupp, 2000; Gurnell, Bertoldi, & Corenblit, 2012; Merritt, Scott, LeROY, Auble, & Lytle, 2010; Toner & Keddy, 1997).

The impact of flow regime variability on riparian vegetation has been well documented in previous literature, such as the detriment when vegetation is scoured (Bendix, 1999; Crouzy, Edmaier, Pasquale, & Perona, 2013; Edmaier, Burlando, & Perona, 2011), covered by sediment or debris (Ballesteros Cánovas et al., 2011), or drowned (Friedman & Auble, 1999), or when the plants’ roots lose their connection to the water table due to channel displacement (Loheide & Booth, 2011). A less obvious example of the negative impact is the general decrease in the vegetation's vigor associated with the poststress reaction of plants. Plants under flood‐induced stress experience both short‐ and long‐term physiological and morphological responses, such as root mortality or reduction in photosynthetic activity, plant growth, and reproduction. Nevertheless, floods can also positively influence riparian vegetation by generating new germination sites, distributing propagules and woody debris (Bertoldi, Gurnell, & Drake, 2011; Gurnell et al., 2012), and enabling access to water and nutrients that cannot usually be reached. Many researches have provided insights into the relative sensitivities of various vegetation groups to riverine flow variation (Bendix & Hupp, 2000; Hupp & Osterkamp, 1985; Johnson, Swanson, Grant, & Wondzell, 2000; Lite, Bagstad, & Stromberg, 2005; Mahoney & Rood, 1998; Osterkamp & Hupp, 1984). These studies have generally considered flood inundation and the groundwater table to be the basic variables that affect individual growth of certain vegetation species, and subsequently influence the structure of the community by controlling soil moisture, nutrient supply, and gas exchange (Camporeale & Ridolfi, 2006; Kozlowski & Pallardy, 1984; Naumburg, Mata‐Gonzalez, Hunter, Mclendon, & Martin, 2005).

There have been many field studies, and many modeling methods proposed focusing on the distribution pattern of riparian vegetation during river flow disturbances. For example, the recruitment box concept (Mahoney & Rood, 1998) described the properly germinating regimes under certain geomorphological features of riverine corridors. The species sorted along water‐depth gradients and stream power gradients were also reported in Bendix and Hupp (2000). A simulating and predicting model was developed by Benjankar, Egger, Jorde, Goodwin, and Glenn (2011), which coupled the digital elevation model of riparian corridors and the fuzzy decision rule of community succession. However, flow fluctuations and physiological water requirements of vegetation can hardly be traced precisely or modeled completely because of the complexity of hydro‐ecological processes.

Recently, the stochasticity in environmental alteration has been widely exploited in modeling methods to further elucidate the spatial–temporal dynamics of vegetation. Most models concentrated on the equilibrium state of the vegetation distribution, although their start point is based on a stochastic process. For example, the maximum information entropy (MaxEnt) principle has been applied in ecology (Harte & Newman, 2014; Harte, Zillio, Conlisk, & Smith, 2008), which deduced species–area relationships and the species abundance distribution in the community, with the constraints on state variables, such as the mean individual number, total energy requirements, and spatial abundance (Harte & Newman, 2014; Harte et al., 2008; Xiao, McGlinn, & White, 2015). These constraint equations represent the spatial equilibrium state of an ecosystem in macroscale geomorphology. Another widely used model, the neutral theory, also involves a stochastic model. Its essential idea assumes that all individuals in a community are strictly equivalent with respect to reproduction and death. This model can simulate the distribution mechanisms behind species abundance patterns in small‐scale research.

The riparian environment is a dynamic ecosystem, in which river flow fluctuation plays a key role in influencing vegetation dynamics (Balke, Herman, & Bouma, 2014; Vesipa, Camporeale, & Ridolfi, 2017). Researchers have developed a series of minimalistic mathematical models with stochastic differential equations (Camporeale & Ridolfi, 2006; Doulatyari, Basso, Schirmer, & Botter, 2014; Perona, Molnar, Savina, & Burlando, 2009; Tealdi, Camporeale, & Ridolfi, 2011, 2013; Tron, Laio, & Ridolfi, 2014; Tron et al., 2015; Vesipa, Camporeale, & Ridolfi, 2015, 2016). Environmental disturbances, such as river flow stage, and flood inundation time, are treated as explanatory variables, and the properties of the vegetation distribution patterns, such as in vegetation biomass, are treated as response variables. The stochasticity involved in disturbances is transformed to random noises mathematically. Most of these models start from two differential equations, which are randomly switched depending on whether the vegetation plot is inundated or exposed (Vesipa et al., 2017), and then the randomly switched differential equations are solved as a stochastic differential equation through mathematical methods like the Fokker–Plank equation and the master equation. The main form of the analytical solution is the plot‐wised probability density function (PDF) of riparian vegetation biomass. These studies are helpful in evaluating flow–vegetation interactions and quantifying the vegetation biomass distribution pattern in a mathematical way. However, few studies can describe the spatial and temporal dynamics of riparian vegetation during river flow disturbances simultaneously, especially the age structure of the vegetation population. The results of the above‐mentioned models are normally deduced from a temporal limit solution of randomly switched differential equations, so that the temporal information in the dynamics system is partly omitted.

The variations in seasonal precipitation, climate, and human water demand change the river flow regime. The natural flow regime and regulated river system would display a significant nonstationarity if their time series were treated as a stochastic process. Plants have varying requirements with respect to the flow regime and tolerances to flood or drought during their life cycle. Only the individuals surviving the effects of the current flow regime can grow up to the next life stage. In general, mature individuals are better able to resist flood inundation and to survive low groundwater levels than young individuals (Friedman & Auble, 1999; Lytle & Merritt, 2004), and seeds have more rigorous requirements for recruitment and germination. Seedling establishment, which usually occurs in specific seasons with suitable hydrologic conditions, is a critical stage in the plant life cycle. This stage determines the scale of vegetation recovery or succession in the subsequent stages. Previous models failed to involve the time‐varying property of the plants’ growing process. There have been few specialized studies on the coupled mechanisms between plant life‐cycle stages and nonstationary river flow regimes, with the exception of some conceptual or qualitative models (Balke et al., 2014; Greet, Angus Webb, & Cousens, 2011; Magurran, 2007; Mahoney & Rood, 1998).

In this work, we formulate a minimalistic and complete stochastic model focusing on the vegetation age structure and describe the spatial–temporal dynamics of riparian vegetation driven by a stochastic river flow process. The mathematical forms in this model are essentially stochastic differential equations with explicit physical meaning. They are solved into stochastic process solutions. The stochastic process solutions and the PDF derived from them describe the dynamic successive processes of a same‐aged population. This model interprets the PDF of abundance rather than vegetation biomass. This study also discusses the calibration and application of the model and deduces the species‐specific age structure with the aid of the Monte Carlo method. This model also has potential to simulate and predict vegetation dynamics when the parameters are calibrated.

## 2 MODEL DESCRIPTION

The purpose of this work is modeling the spatial–temporal dynamics of riparian vegetation using a process‐based minimalistic mathematical method. In order to achieve this, we focus on a species‐specific population of the same age, rather than vegetation biomass. A same‐aged population, from its seedling germination to extinction, will experience a series of life stages, such as seedling germination, establishment, growth, aging, and mortality. The adaptability and tolerance of this population to the river flow regime are time‐varying with the individuals’ growth. Seeds may successfully colonize and finally the population may develop to a persistent vegetation cover if there are proper flow regimes in the riparian corridors. On the contrary, seedlings may occasionally sprout but fail to survive under extreme hydrologic disturbances at a certain life stage, so that the seedling population may demonstrate expansion or reduction dynamics under different conditions. A mature population will only decrease because of detrimental disturbance and will not expand under suitable conditions, as a seedling population does.

For a same‐aged population in formative stages, such as seedling germination and establishment, the deterministic model describing its dynamics is the logistic equation, while for a population in declining stage, the deterministic model is the exponential decay equation. Thus, the minimalistic deterministic model of the dynamics of a same‐aged population becomes:

(1) $$\frac{dn}{dt}=\left\{\begin{array}{cc} r\cdot n(1-n), & t<T_{f},\\ -r\cdot n & t>T_{f}, \end{array}\right.$$

where n represents normalized abundance of the vegetation population, $T_{f}$ is duration length of formative stage, and r is the population growth rate.

**Figure 1:**
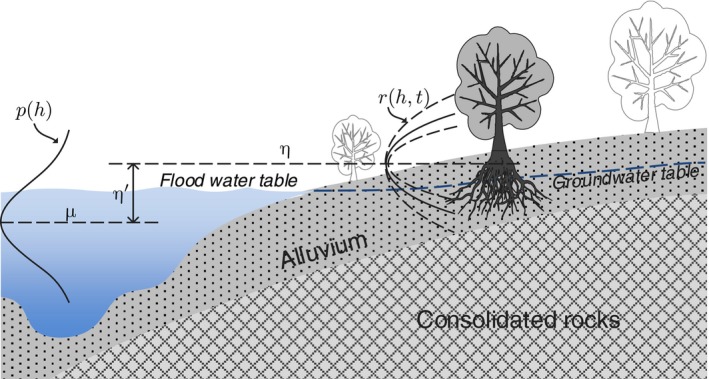
Sketch of riparian corridor transect and the main variables in the minimalist stochastic model. h is the river flood water level. p(h) represents the probability density of the flood water level with its mean μ. η represents the plot‐wised favorable level for the species‐specific individuals. r(h,t) illustrates the population growth rate versus h and time t

While the population growth rate of riparian vegetation is hydrology dependent, when considering the stochasticity of river flow fluctuations, the population growth rate becomes a random variable depending on the river flow regime. Because extreme hydrological conditions may lead to individual death and population decline, riparian plants generally prefer a moderate flood water table, and any departure from the favorable water table will decrease the population growth rate. If the water table declines from the favorable level, xylem cavitation, stomatal closure, and emboli may occur, which lead to decreased photosynthesis (Cooper, Dámico, & Scott, 2003; Sperry, Adler, Campbell, & Comstock, 1998); or if the water table rises from the favorable level, gas exchange in roots will reduce and anoxia will occur (Bogino & Jobbágy, 2011; Naumburg et al., 2005). The favorable water table η to a population in a plot will induce a maximum growth rate (Figure 1). So that the random growth rate r, as a function of random water table h, can be written as:

(2) $$r\left(h\right)=\left\{\begin{array}{cc} \lambda -a_{f}\cdot D\left(h-\eta \right) & t<T_{f},\\ -a_{d}\cdot D\left(h-\eta \right) & t>T_{f}, \end{array}\right.$$

where λ represents the maximum growth rate under favorable water table, D(·) is a divergency metric function of the water table departure from the favorable level, $a_{f}$ and $a_{d}$ are the sensitivity coefficients of population to the disturbances. λ has a positive value, which means the vegetation population in a formative stage may expand under favorable conditions. The divergency metric function is concave and its maximum zero lies in η=h. The simplest metric function is quadratic (see also Phipps (1979); Pearlstine, McKellar, and Kitchens (1985); Camporeale and Ridolfi (2006)) as $D\left(h-\eta \right)\;=\;{\left(h-\eta \right)}^{2}$.

During the life cycle of a population, its sensitivity to the flood water table is time‐varying. We model $a_{d}$ as a time‐dependent function in which both the growing processes and the senescence processes are considered. It can be written as follows:

(3) $$a_{d}\left(t\right)=A+g_{1}\left(t\right)+g_{2}\left(t\right),$$

where constant A is a parameter to guarantee continuity, $g_{1}\left(t\right)$ reflects the sensitivity reduction with growing, and $g_{2}\left(t\right)$ models the sensitivity increase with aging. $T_{f}$ is duration length of the formative stage and $a_{d}\left(T_{f}\right)$ can be set to $a_{f}$ to maintain continuity at time $T_{f}$. An example of $a_{d}\left(t\right)$ is written as:

(4) $$\left\{\begin{array}{c} g_{1}\left(t\right)=B/t,\\ g_{2}\left(t\right)=Ce^{\mathrm{Dt}}, \end{array}\right.$$

where $g_{1}\left(t\right)$ reflects the linear growth of individual height, and $g_{2}\left(t\right)$ simulates the exponential aging process after the growth inflection point, and B, C and D are undetermined coefficients. Then, the four parameters of $a_{d}\left(t\right)$, including A, B, C and D, can be calibrated according to growing models from which the following conditions can be deduced: (1) the turning point between growing and aging processes; (2) the minimum sensitivity of mature individuals; and (3) the species‐specific aging velocity. $a_{f}$ is assumed to be a constant for the relatively short duration of the formative stage during which the individual amount rather the volume displays significant variation. While in the decline stage, $a_{d}$ is a time‐dependent function in which both the growing processes and the senescence processes are considered. Because the parameters like $a_{f}$, $a_{d}$, λ and η are species specific, so the stochastic model is species specific.

### 2.1 Stochastic fluctuation of water table

The fluctuation of the flood water table h is affected by various physical processes within the river system, including river discharge, precipitation, evaporation, and anthropogenic regulations (Poff et al., 1997). The probability density and time‐correlation function of h are the two main important functions to theoretically describe this stochastic fluctuation. However, it is difficult to determine the exact probability density function (PDF) of h. Camporeale and Ridolfi (2006) use a single‐parameter Gamma distribution as an approximation of the PDF of flood water table. We assume the Gaussian density for h based on two considerations. First, there are numerous complex and independent mechanisms that affect riverine physical processes. Second, in the river basin, the effects of channels and dams on the river discharge are analogous to the effects of a capacitor in an electronic circuit; this means that the water table is comparable to voltage noise, which has been proven to follow a Gaussian distribution (Jacobs & Wozencraft, 1965). We have performed a goodness of Gaussian density fit test on the hydrology data recorded by three hydrometric stations of China, and results verified the Gaussian density assumption is acceptable.

The correlation time represents how fast the flood water table changes. If river flow changes faster than the vegetation population, the correlation time of the changing river flow can be neglected, then h can be treated as a white noise process. This holds for both the formative stage and decline stage of the same‐aged population if h experiences fast fluctuation. Otherwise, if the river flow changes more slowly than the vegetation population, the correlation time must be considered. The Ornstein–Uhlenbeck (O‐U) process is usually used to model stochastic processes with non‐negligible correlation time (Arnold, Horsthemke, & Lefever, 1978; Kitahara, Horsthemke, Lefever, & Inaba, 1980; Van Den Broeck, 1983). And this situation usually holds for the formative stage of populations because relatively fast variation in seedling communities. The timescale of flood water table variation is typically several weeks to several 100 days (Balke et al., 2014; Richter, Baumgartner, Powell, & Braun, 1996). However, the typical timescale of same‐aged population evolution generally ranges from several days to several months in the formative stage, and from several years to decades (Camporeale, Perucca, Ridolfi, & Gurnell, 2013) in the decline stage. Because the difference between temporal scales of river flow changes and mature population vegetation dynamics are ordinarily several orders of magnitude, we can model the stochastic fluctuation of the water table as white Gaussian noise (W.G.N.) when studying population dynamics in the decline stage, while, when studying the population in the formative stage, we have to consider whether the flow changes faster than the population evolves or not. Assuming the correlation time of h is $\tau_{h}$, h can be written as follows:

(5) $$h\left(t\right)≜\mu +\sigma e^{-\alpha t}B\left(e^{2\alpha t}\right),$$

where $\alpha \;=\;1/\tau_{h}$ if $\tau_{h}$ cannot be neglected, where μ and σ are the mean and standard variance of h, or

h(t)≜W.G.N.

if $\tau_{h}$ can be neglected.

### 2.2 Analytical solution

After involving the stochasticity of h(t), Equation (1) becomes a stochastic differential equation. Because the equation and fluctuations of h have clear physical meaning, in this work, we use the Stratonovich calculus (Risken, 1989; Van Kampen, 1981) to obtain the analytical solution. Introduce variables $h^{\prime }\left(t\right)\;=\;h\left(t\right)-\mu$ as the variation part of h(t) and $\eta^{\prime }\;=\;\mu -\eta$ as the divergency of the water table, expand Equation (1), and rewrite it as follows:

(6) $$\frac{dn}{dt}=\left\{\begin{array}{cc} \left[\lambda -a_{f}\left(\eta^{\prime 2}+h^{\prime 2}+2\eta^{\prime }h^{\prime }\right)\right]\cdot n\left(1-n\right), & t<T_{f},\\ -a_{d}\left(\eta^{\prime 2}+h^{\prime 2}+2\eta^{\prime }h^{\prime }\right)\cdot n & t>T_{f}, \end{array}\right.$$

Using the variable substitution following Stratonovich rule, there is:

$$\left\{\begin{array}{cc} \frac{d}{dt}\text{logit}\left(n\right)=\lambda -a_{f}\left(\eta^{\prime 2}+h^{\prime 2}+2\eta^{\prime }h^{\prime }\right), & t<T_{f},\\ \frac{d}{dt}\log\left(n\right)=-a_{d}\left(\eta^{\prime 2}+h^{\prime 2}+2\eta^{\prime }h^{\prime }\right), & t<T_{f}. \end{array}\right.$$

For the case of h(t)≜W.G.N., we can get:

(7) $$\left\{\begin{array}{cc} \text{logit}\left(n\right)=c_{1}+\left(\lambda -a_{f}M\right)t+2a_{f}\Sigma B\left(t\right), & t<T_{f},\\ \log\left(n\right)=c_{2}+M\int_{T_{f}}^{t}a_{d}\left(t\right)dt+2\Sigma \int_{T_{f}}^{t}a_{d}\left(t\right)dB\left(t\right), & t>T_{f}, \end{array}\right.$$

in which B(t) represents the normal Brownian motion, $c_{1}$ is the initial value $\text{logit}(n_{0})$, $c_{2}$ is a constant to maintain continuity, and M and Σ are substitution variables as follows:

(8) $$\begin{array}{cc} M & =\eta^{\prime 2}+\sigma^{2},\\ \Sigma & =\eta^{\prime }\sigma . \end{array}$$

The above solution is also a stochastic process, which involves the representation of Brownian motion. This means logit(n) and log(n) are some kind of Gaussian process, and they have Gaussian PDF. The PDF of logit(n) can also be solved using the Fokker–Plank equation method. From Equation (7), we can get the mean and variance of logit(n) and log(n) as follows:

(9) $$\begin{array}{cc} & \begin{array}{cccc} \;\;\; & \text{mean}\;\; & \text{variance} & \; \end{array}\\ & \{\begin{array}{cccc} \text{logit}(n): & c_{1}+\left(\lambda -a_{f}M\right)t & 4a_{f}^{2}\Sigma^{2}t & t<T_{f},\\ \log(n): & c_{2}+M\int a_{d}\left(t\right)dt & 4\Sigma^{2}\int a_{d}^{2}\left(t\right)dt & t>T_{f}. \end{array} \end{array}$$

For the case of h(t)≜O‐Uprocess, the mean and variance of logit(n) are calculated based on the above idea:

(10) $$\begin{array}{cc} & \text{mean:}\;c_{1}+\left(\lambda -a_{f}M\right)t,\\ & \text{variance:}\;V_{1}+V_{2}+V_{3}, \end{array}$$

where

(11) $$V_{1}=4a_{f}^{2}\Sigma^{2}t,$$

(12) $$V_{2}=\frac{8a_{f}^{2}\Sigma^{2}}{\alpha^{2}}\left(\alpha t-1+e^{-\alpha t}\right),$$

(13) $$V_{3}=\frac{a_{f}^{2}\sigma^{4}}{\alpha^{2}}\left(2\alpha t-1+e^{-2\alpha t}\right).$$

Term $V_{1}$ is equal to the variance of logit(n) in the case of h(t)≜W.G.N. $V_{2}$ and $V_{3}$ are additional terms brought by correlation time $\tau_{h}$. Among them, $V_{2}$ is related to both the flood flow parameters $\eta^{\prime }$ and σ through Σ, but $V_{3}$ is only related to σ.

## 3 RESULTS

### 3.1 Abundance of the population

**Figure 2:**
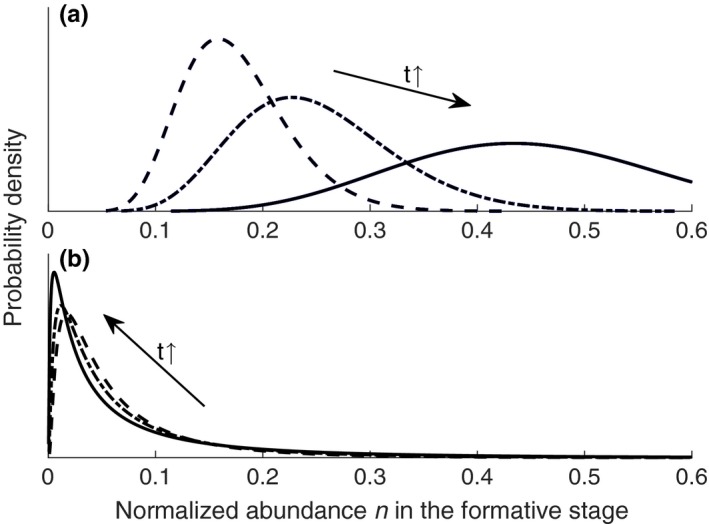
Probability density of the normalized abundance in the formative stage. (a) and (b) shows the probability density function of normalized abundance and the curves correspond to different evolution time. (a) represents a vegetation sample plot with small divergency $\eta^{\prime }$, so the population shows a increasing tendency in the formative stage. While (b) represents a sample plot with great divergency, so that the population shows a decreasing tendency

Presume the initial value is n(0)=ϵ, which is a small positive number that represents the occasional germination (e.g., 0.05) when the population evolution starts. From the solution (7), the logit function of n in formative stage and log function of n in declining stage both follow normal distribution. Therefore, the abundance of a same‐aged population gradually transitions from following logit normal distribution to following log‐normal distribution. In the formative stage, whether the population will expand or shrink is determined by the sign of term $\lambda -a_{f}M$, Figure 2 shows the PDF evolution in formative stage within two vegetation plots with different divergency $\eta^{\prime }$. Figure 2(a) illustrates a vegetation plot with small divergency $\eta^{\prime }$, the same‐aged population shows a increasing tendency; and Figure 2(b) illustrates a vegetation plot with great divergency, so that the population shows a decreasing tendency. In the declining stage, the population will always display a decreasing tendency. The distribution of total vegetation abundance within a riparian area will be a composition of a series of logit normal distributions and log‐normal distributions. Because the logit normal distribution is left‐inclined relative to log‐normal distribution, the distribution of vegetation abundance will also hold this feature (see also Chave (2004); Magurran (2007)).

Equation (8) shows that the standard deviation of flow fluctuation σ and water table divergency $\eta^{\prime }$ play symmetric roles in shaping the PDF of population n when the riverine flow regime experiences a rapid alteration relative to the evolution of the seedling population. It will be different when the river flow experiences slow alteration.

### 3.2 Effect of correlation time

**Figure 3:**
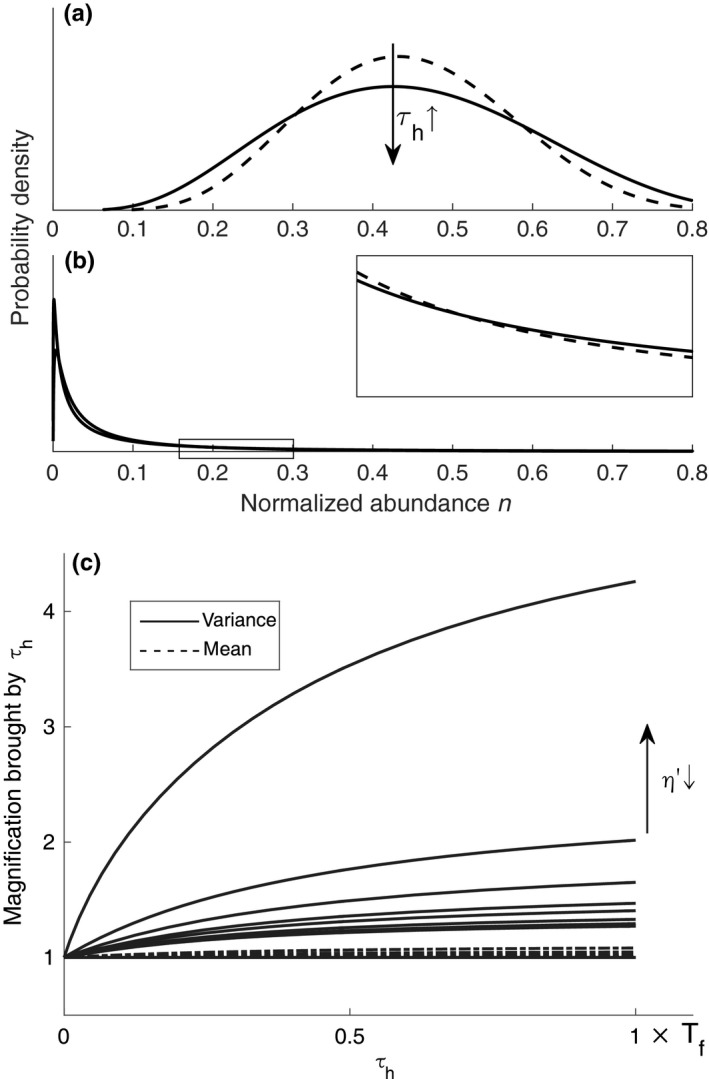
Effects of the correlation time $\tau_{h}$ of river flow changes on the mean and variance of population abundance in formative stage. (a) and (b) illustrates the effects on the probability density functions of abundance in two sample plots with different $\eta^{\prime }$. In both (a) and (b), $\tau_{h}$ increases the variances, that is, increases the probability of extremely high and low abundance (solid line). (c) The correlation time $\tau_{h}$ magnifies both the mean (dashed lines) and variance (solid lines), and the magnifications on variance are much greater than the magnifications on mean

The correlation time $\tau_{h}$ reflects the river flow change rate. A large $\tau_{h}$ means river flow regime changes slowly, and the detrimental and favorable flow regimes will remain stable for a relatively long duration. The effects of the correlation time $\tau_{h}$ on the distribution of n should be considered then. Equation (7) shows that $\tau_{h}$ increases the variance of logit(n) but has no effect on its mean. Nevertheless, $\tau_{h}$ can increase both mean and variance of n because of the nonlinearity inverse transformation of logit(n) (Figure 3c). $\tau_{h}$ generally affects the variance of n more strongly than the mean. A non‐negligible correlation time $\tau_{h}$ increases the probability of extremely high and low vegetation abundance (Figure 3a,b). Meanwhile, the standard deviation σ and the divergency $\eta^{\prime }$ do not have equivalent roles on vegetation distribution; σ, with the aid of $\tau_{h}$, contributes an additional term to the variance of logit(n) (Equation (13)).

If the population in the formative stage, such as seedling germination and establishment, experiences a favorable river flow regime, it will have more opportunities to expand its size and survive future detrimental flow regimes. Otherwise, if it encounters detrimental flow regime in its early life stage, many individuals in it will lose opportunities to survive until the future favorable conditions occur. This indicates that river flow regulation can be effective for the conservation and recovery of riparian vegetation. Gherardi and Sala (2015) came to a similar conclusion through experiments showing that precipitation variance had a positive effect on relative vegetation abundance and functional diversity.

### 3.3 Population lifespan

**Figure 4:**
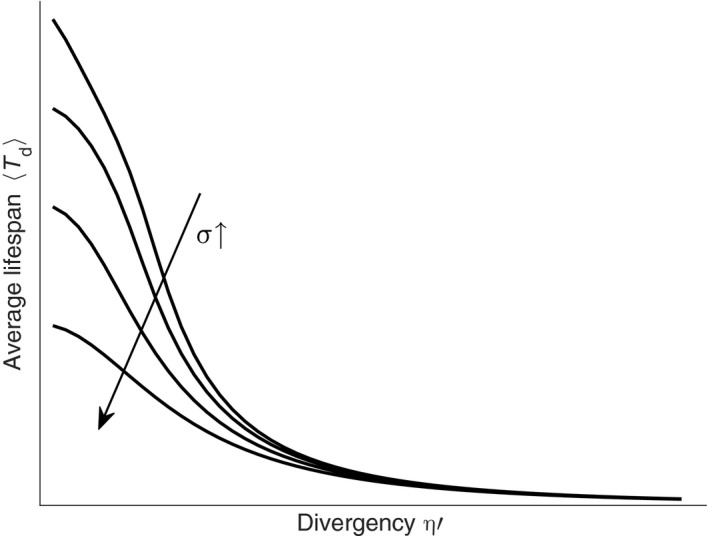
Average lifespan $\left\langle T_{d}\right\rangle$ of the same‐aged population versus the divergency $\eta^{\prime }$. The curves correspond to different deviation σ. $\left\langle T_{d}\right\rangle$ generally displays a decreasing tendency as divergency $\eta^{\prime }$ increasing, which means that population in the vegetation plot with favorable flow conditions will have relatively long lifespan. The deviation σ can induce greater lifespan difference in sample plots with favorable flow conditions than in plots with unfavorable conditions

After the seedling establishment season ends, no more seeds will germinate, and seedling grow up to mature individuals gradually. Equation (7) shows that logarithmic abundance log(n) of the same‐aged population is a Gaussian diffusion process with a decreasing mean and increasing variance over time. Presume when n(t) is smaller than a lower limit $n_{e}$ (e.g., a value smaller than ϵ), all individuals within this same‐aged population are deemed to be extinct and the population disappears. The time $T_{d}$ from germination of seedlings to the population's eventual disappearance is a stopping time according to the stochastic process theory, and it has a complex probability distribution because of time‐varying drift rate and diffusion coefficient. While the mean of $T_{d}$ can be obtained from the stopping theorem as follows:

(14) $$\left\langle m_{2}\left(T_{d}\right)\right\rangle =\left\langle m_{2}\left(T_{f}\right)\right\rangle -M\int_{T_{f}}^{\left\langle T_{d}\right\rangle }a_{d}\left(t\right)dt,$$

where $\left\langle m_{2}\left(T_{d}\right)\right\rangle$ is the lower limit $n_{e}$, then the average lifespan $\left\langle T_{d}\right\rangle$ could be solved. If the population is germinated in a plot with comfortable flow conditions, that is, with small divergency $\eta^{\prime }$, the rate of population decaying is also small, the alterations of $\eta^{\prime }$ and σ will deduce large lifespan intercepts (Figure 4). But for the plots with severe flow conditions, the alterations of $\eta^{\prime }$ and σ will deduce small lifespan intercepts although the lifespan is indeed decreased by those severe flow conditions. Because $\eta^{\prime }$ and σ have symmetric position in the Equation (14), the curves of average lifespan $\left\langle T_{d}\right\rangle$ versus σ will display the same shape with above figure.

### 3.4 Vegetation distribution along riparian transect

To better understand the spatial dynamics of riparian vegetation, we study the distribution patterns of both average abundance and lifespan along the direction perpendicular to river runoff. From Equations (7) and (14), the average abundance and the average lifespan display similar dependence on $\eta^{\prime }$ and σ, that is a monotonic decrease tendency over σ and a single‐peaked tendency over $\eta^{\prime }$ with a peak at $\eta^{\prime }\;=\;0$, which represents the most favorable colonized location. However, a higher ground surface elevation does not obviously induce a larger difference between the required and actual groundwater table. Divergency $\eta^{\prime }$ and deviation σ of the flow water table are both dependent on the location x.

The existence of soil and the capillary fringe can resist part of the increase in divergency $\eta^{\prime }$ between elevated land surface and favorable water table. Meanwhile, groundwater variation is also weakened by soil and the capillary fringe, and this corresponds to a smaller deviation σ of h. Therefore, the river flow parameter pair $\left(\eta^{\prime },\sigma \right)$ can be linked to the geomorphological features or the geographical coordinates.

**Figure 5:**
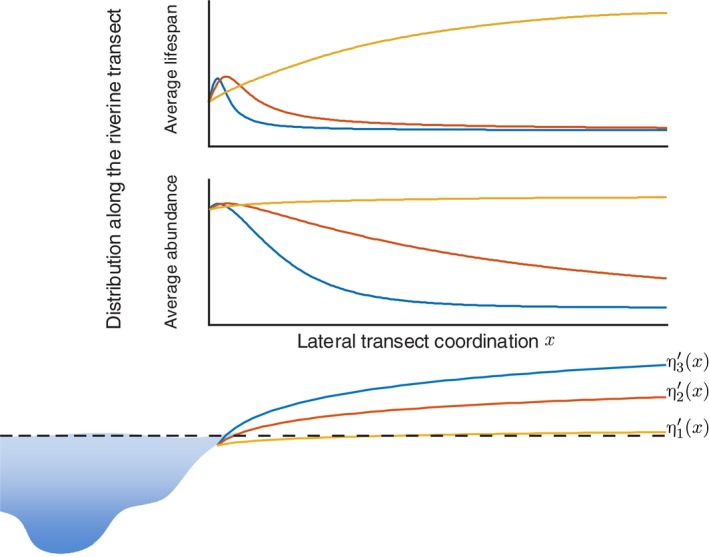
Illustration of distribution patterns along the riparian transect. Different $\eta^{\prime }\left(x\right)$ and σ(x) can induce various distribution patterns of vegetation abundance and lifespan. In order to investigate the distribution patterns, three logarithmic divergency gradients along the direction of riparian transect are assigned, and three different patterns (color coded curves) of vegetation abundance and lifespan can be deduced corresponding to the different gradients. These patterns reflect their dependency on the geomorphology of riparian area

We assume that σ decreases linearly with x because the change of saturated water table is less related to river flow fluctuation with x increasing. Then, the distribution patterns of the average abundance $\left\langle n\left(T_{f}\right)\right\rangle$ and average lifespan $T_{d}$ versus the distance x were investigated using three $\eta^{\prime }\left(x\right)$, which represent slow, moderate, and rapid decline of the saturated water table with distance x, respectively. The result shows three different patterns for both average abundance and average lifespan: (1) monotonically increasing pattern; (2) peaked pattern but relatively flat after the peak; and (3) peaked pattern but rapidly declining after the peak (Figure 5). To date, there are few field data on the lifespan distribution of riparian vegetation, except for the age investigation of cottonwoods by Mahoney and Rood (1998). Their survey data Mahoney and Rood (1998) confirmed the first pattern of vegetation lifespan.

The first abundance pattern of monotonically increasing tendency was found by Bradley and Smith (1986) for the Milk River (Canada) and by Brookes, Hooke, and Mant (2000) for the Mediterranean region. The second pattern of single‐peaked and slowly decreasing tendency was found by Carter Johnson, Dixon, Simons, Jenson, and Larson (1995), Naiman et al. (2010) and Lytle and Merritt (2004), and the left clining PDF relative to log‐normal distribution was also supported by a detailed investigation based on a tree census in a moist tropical forest (Chave, 2004). The third pattern of single‐peaked but rapidly decreasing tendency is supported by the studies of some riparian areas within semiarid or arid regions (Carr, 1998; Tooth, 2000). These studies showed that various types of tree and forb had similar distribution shapes. The STEM presents that this performance of abundance of a similarly aged population holds over its entire lifetime.

## 4 DISCUSSION

This study aimed to theoretically model the spatial and temporal dynamics of riparian vegetation during river flow fluctuations. We focused on how the same‐aged population of specific species evolves under flood water disturbances. The minimalistic stochastic model developed in this paper deduces a process‐based solution, which is helpful for studying the vegetation temporal dynamics, lifespan, and even age structure. Our main achievements are as follows:

First, we find the distribution of the vegetation population can be described as a combination of logit normal and log‐normal. Second, the result about temporal dynamics of the same‐aged population indicates that the relatively favorable flow condition is particularly important for younger populations. A population will grow better when it experiences relatively comfortable flow conditions followed by relatively severe conditions, than in the reverse situation. Third, the model quantifies the effect of the correlation time of river flow fluctuation. River flow regulation and water resource scheduling for vegetation recovery and conservation is supported by the results of our model. Fourth, the model can deduce different patterns for vegetation abundance and lifespan, which shows it can describe vegetation spatial dynamics.

Stationarity of the stochastic river flow fluctuation is not an intrinsic requirement in the model, because the model and its results are both process‐based. The nonstationarity of river flow fluctuation is generally induced by seasonal or annual river discharge, precipitation changes, and human extractions. If the river flow displays significant nonstationarity, it can be divided into several relatively stationary segments, and then the riparian vegetation dynamics can be analyzed by segment.

Steiger, Tabacchi, Dufour, Corenblit, and Peiry (2005) summarizes the factors that can influence riparian vegetation dynamics. Although previous studies of riparian vegetation systems have included a rich quantity of flow and vegetation data, it is difficult and time‐consuming to build a complete dataset, as listed in figure 4of Steiger et al. (2005), based on samples from many locations. The results of this work are qualitatively verified by the field investigations in previous studies.

### 4.1 Calibration

There are two types of parameter in the model: river flow parameters and species parameters. These parameters can be calibrated using actual records of hydrology and field investigation of riparian vegetation. The river flow parameters, such as μ, σ, and $\tau_{h}$, can be estimated according to hydrological records. The species parameters, such as A and B, C and D in Equation (3), can be fitted with plant growth data or plant growth models, such as JABOWA (Botkin, Janak, & Wallis, 1972; Pearlstine et al., 1985; Shugart & West, 1977). The calibration equations can be written as follows:

$$\begin{array}{cc} a_{d}\left(T_{f}\right)=a_{f}, & \mathrm{continuity},\\ \dot{a_{d}}\left(t_{\mathrm{ip}}\right)=0, & \mathrm{growth}\;\mathrm{inflection},\\ a_{d}\left(t_{\mathrm{ip}}\right)=\min\left(a_{d}\right), & \mathrm{minimum}\;\mathrm{sensitivity},\\ a_{d}\left(t_{\max\;\mathrm{age}}\right)=\max\left(a_{d}\right), & \mathrm{maximum}\;\mathrm{sensitivity}, \end{array}$$

where $t_{\mathrm{ip}}$ and $t_{\max\;\mathrm{age}}$ represent inflection age and maximum age of the species, respectively.

The other species parameters $a_{f}$, λ, and η should be calibrated using field sample data or experimental data. For example, presume we have more than three sample plots (Figure 7a) located at the riparian transect and river flow parameters are fitted. First, we can get $\lambda -a_{f}M$ using the sample value of logit(n) and log(n) according to least square method and Equation (9). The calibration equations of the undetermined parameters can be written as follows:

$$\begin{array}{cc} \lambda -a_{f}M_{1}=v_{1}, & \text{plot 1}\\ \lambda -a_{f}M_{2}=v_{2}, & \text{plot 2}\\ \vdots & \vdots \\ \lambda -a_{f}M_{n}=v_{n}. & \text{plot n} \end{array}.$$

The favorable water table η is also a plot‐wised parameter because it is location dependent, so that we have:

$$\eta_{i}-\eta_{j}=\eta_{i}^{\prime }-\eta_{j}^{\prime }={\text{ALT}}_{i-j},$$

where ${\text{ALT}}_{i-j}$ represents the altitude difference between plot i and j. Then, the parameters can be solved.

### 4.2 Simulation

**Figure 6:**
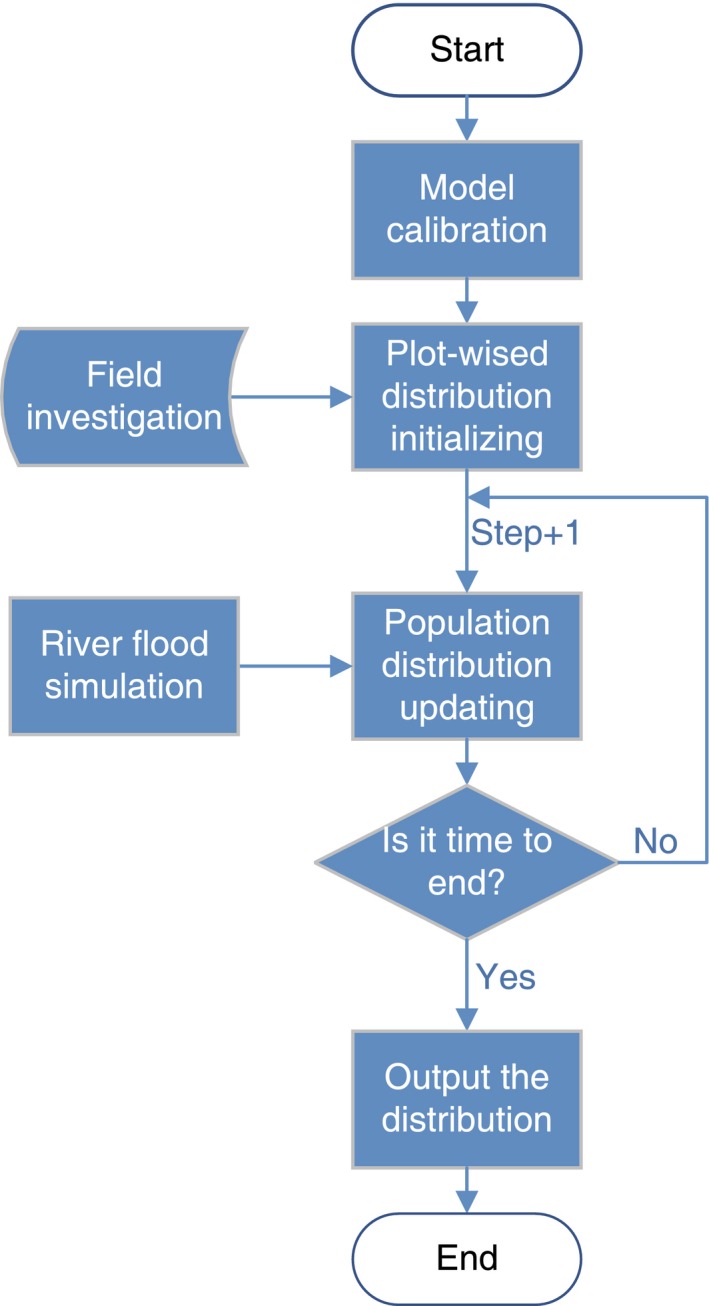
Flowchart for simulation and prediction. First of all, the parameters calibration is needed based on the current or history distribution pattern, hydrological data, and geomorphological model. The output of every running loop is also distribution pattern

**Figure 7:**
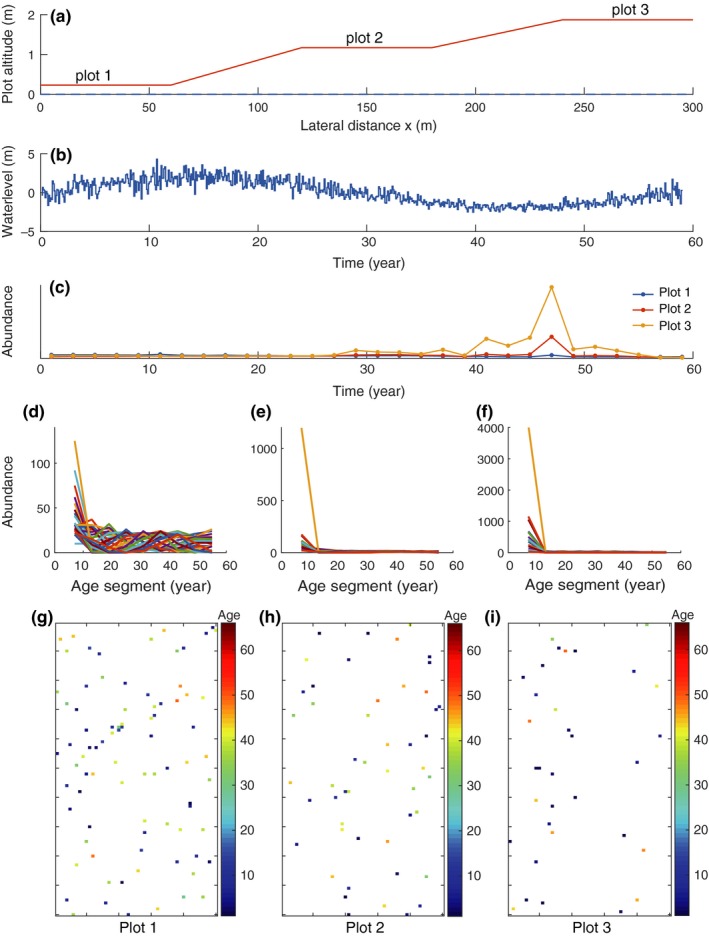
Simulation of three presumed white birch plots. (a) Illustration of three white birch plots’ position along the riparian transect. (b) The simulated river flow regime: a long‐term flow alteration in 60 years. (c) The abundance dynamics of the three plots. The alteration of flow regime induces lagging alteration of vegetation abundance. (d–f) shows the age structure alteration of the three plots, respectively. (g–i) illustrates the final pattern of the three plots at the end of simulation

After all the parameters are calibrated, the model can be easily applied to simulate or predict the dynamics of vegetation because of its process‐based solution. The flowchart is shown in Figure 6. The simulation or prediction must start from calibration of all plot‐wised parameters and inputting geomorphological data across the studied riparian region. Using field data sampled from three plots of white birch forest located in a Luan river sample area in China, we calibrated river flow parameters and species parameters for three sample plots of white birch as listed in Table 1. Figure 7a illustrates a simulated transect of riparian area with three sample plots. Then, we presume a long‐term alteration of the river flow regime (Figure 7b) as driving process of the dynamics of three simulated white birch plots. This simulates a case that the mean and variance of flood water table experience a sine‐like drift in 60 years. $\tau_{h}\;=\;0$ represents a relatively fast intermonthly fluctuation. Figure 7c shows the dynamics of total not same‐aged abundances. From the simulation results, the abundance display lagging alteration behind river flow. And because three plots’ altitudes are different, the favorable water condition are not obtained simultaneously by three plots, so that the abundance order of three plots reverses at about thirtieth years. Figure 7d–f shows the age structure alteration of three plots in 60 years, from which, we can see that plot 1 has the most even age structure, plot 2 has a younger age structure than plot 1 and plot 3 has the most younger age structure. The abundance peaks of three plots located at about forty‐seventh year (Figure 7c) and the following fast decreasing tendencies indicates a group of young individuals emerge centrally and are destroyed quickly. The age structures also reflect the general flow regime suitability for the species located in different plots. Finally, Figure 7g–i visually presents the distribution patterns of three plots at the last simulation year.

**Table 1:** Calibrated parameters in the application of this model

River flow parameters (m)	Species parameters^a^
μ=82.8	A=−0.024, B=0.513
σ=0.58	$C\;=\;7\;\times \;10^{-4}$, D=0.04
	$\lambda \;=\;3.24,\;a_{f}=0.49$
	Plot 1: $\eta_{1}\;=\;83.03$
	Plot 2: $\eta_{2}\;=\;83.97$
	Plot 3: $\eta_{3}\;=\;84.67$

a The time unit is year.

## 5 CONCLUSION

The model gives an explicit and theoretical framework and adopts temporally segmented stochastic differential equations rather than randomly switched ones. The growth rate of a population, as a unifying link variable between flood water table and riparian vegetation, is comprised of an intrinsic part and a random part. The intrinsic part is determined by the relatively steady macro‐environmental conditions in the riparian area, and the random part is driven by the randomly fluctuating flood water table. This modeling has an explicit physical meaning, and it reflects the temporal dynamics of riparian vegetation better than models in which carrying capacity is treated as a random variable. The effect of correlation time of river flow fluctuation is also quantified in this work. The result indicates that the larger the correlation time of river flow fluctuation is, the greater variation in riparian vegetation distribution. From our theoretical results, reasonable river flow management and regulation would be helpful for riparian vegetation protection and restoration. Based on the Markov property involved in the stochastic differential equation, the model can simulate the spatial–temporal dynamics of a population with the field data as the initial value.

The model framework is open. Factors that have small spatial–temporal scale (Steiger et al., 2005) relative to dynamics of the same‐aged population can be combined into the modeling of growth rate through adding extra drift and diffusion terms. Furthermore, using other specific functions, or even numerical forms fitted from field investigation data, the time‐varying sensitivity coefficient can be redefined, then the model would be more applicable to various species.

## CONFLICT OF INTEREST

None declared.

## AUTHOR CONTRIBUTIONS

Xiaoguang You and Jingling Liu developed and framed research questions. Xiaoguang You developed the model, analyzed the results, and wrote the first draft of the manuscript. All authors contributed to discussing the results and editing the manuscript.
